# Ghrelin and Cardiovascular Diseases

**DOI:** 10.2174/157340310790231662

**Published:** 2010-02

**Authors:** Gaigai Zhang, Xinhua Yin, Yongfen Qi, Lakshmana Pendyala, Jack Chen, Dongming Hou, Chaoshu Tang

**Affiliations:** 1 Cardiology Department, the First Affiliated Hospital of Harbin Medical University, Harbin,P. R. China 150081; 2 Department of Physiology and Pathophysiology, Peking University Health Science Center, Beijing, P. R. China100191; 3 Saint Joseph’s Translational Research Institute/Saint Joseph’s hospital Atlanta, Norcross, GA 30071, USA

**Keywords:** Ghrelin, coronary artery disease, hypertension, cardiomyopathy, heart failure, cachexia.

## Abstract

Ghrelin, a newly discovered bioactive peptide, is a natural endogenous ligand of the growth hormone (GH) secretagogue receptor and initially identified as a strong stimulant for the release of GH. Subsequent research has shown that ghrelin and its various receptors are ubiquitous in many other organs and tissues. Moreover, they participate in the regulation of appetite, energy, bodyweight, metabolism of glucose and fat, as well as modulation of gastrointestinal, cardiovascular, pulmonary, immune functions and cell proliferation/apoptosis. Increasing evidence has demonstrated that ghrelin has a close relationship with cardiovascular system. Ghrelin and its receptors are widely distributed in cardiovascular tissues, and there is no doubt that the effects of ghrelin in the cardiovascular system are mediated not only via its growth-hormone-releasing effect but also by its direct effects on the heart. Exogenous administration of ghrelin can dilate peripheral blood vessels, constrict coronary artery, improve endothelial function, as well as inhibit myocardial cell apoptosis. So, ghrelin may have cardiovascular protective effect, including lowering of blood pressure, regulation of atherosclerosis, and protection from ischemia/reperfusion injury as well as improving the prognosis of myocardial infarction and heart failure. Some of these new functions of ghrelin may provide new potential therapeutic opportunities for ghrelin in cardiovascular medicine. In this paper, we will review the existing evidence for cardiovascular effects of ghrelin, including the cardiovascular function, the variations in ghrelin plasma levels in pathophysiologicalogical conditions, the possible protective mechanisms of ghrelin, as well as its future potential therapeutic roles.

## INTRODUCTION

Ghrelin, a peptide of 28 amino acids, was first reported by Kojima in rat and human stomachs in 1999 [1]. The ripe peptide of ghrelin is cleaved from its precursor preproghrelin and the gene encoding human preproghrelin is located on chromosome 3p25-26 [2]. Then serine-3 of ghrelin is acylated with Ghrelin O-Acyltransferase (GOAT) [3] and this esterification is essential for its activity. Two thirds of the circulating ghrelin originates in X/A-like cells of the oxyntic mucosa of the stomach and rest produced by X/A-like cells of the small intestine [4]. In addition, smaller amounts of ghrelin is also produced by other organs, such as heart, lung, kidney, pancreas, placenta, lymphatic tissue, gonads, thyroid, adrenal, pituitary, hypothalamus, and some neoplastic tissues and cancer-cell lines [2, 5-11].

In healthy adults plasma, the acyl-ghrelin is 10~20fmol/ ml, and the total ghrelin (both acyl-ghrelin and non-acyl-ghrelin) is 100~150fmol/ml [2]. The half-life of ghrelin in circulation is less than 60 min, and ghrelin released from internal organs is very important for maintaining the plasma concentration [12]. While, ghrelin is seen in the cerebral spinal fluid but at much lower concentration, about 1/30-1/40 of that of circulating ghrelin [13].

As the endogenous ligand of the growth hormone secretagogue receptor (GHS-R), ghrelin was initially identified to be a strong stimulant for the release of GH [14]. In subsequent research ghrelin and its various receptors were found ubiquitous in many organs and tissues. Moreover, they participate in the regulation of appetite, energy, bodyweight, metabolism of glucose and fat, as well as modulation of gastrointestinal, cardiovascular, pulmonary and immune functions, cell proliferation/apoptosis [15, 16]. Although ghrelin and GHS-R1a knockout animals look similar in appearance to wild-type animals, may suggesting that ghrelin does not play a essential role in growth and development [17], both administration of exogenous ghrelin and ghrelin gene over expression exhibits increased circulating ghrelin accompanied with increased bioactive effect or some therapeutic effects. In this paper, we will review the existing evidence for cardiovascular effects of ghrelin, including the cardiovascular function, the variations in ghrelin plasma levels in pathophysiologicalogical conditions, the possible protective mechanisms of ghrelin, as well as its future potential therapeutic roles.

## CARDIOVASCULAR DISTRIBUTION OF GHRELIN AND ITS RECEPTORS

As a cardiovascular hormone, ghrelin and its receptors widely exist in cardiovascular system. Maria and his co-investigators found the expression of ghrelin mRNA in cultured HL-1 cell line (a kind of adult mouse cardiomyocyte) by reverse transcription polymerase chain reaction (RT-PCR), and the expression of ghrelin peptide in HL-1 and human cardiomyocytes by immunohistochemistry and in the cultured medium of these cells by radioimmunity assay [18], suggesting ghrelin may exert paracrine/autocrine effects in cardiovascular system. Gnanapavan and colleagues found the expression of ghrelin mRNA in human atrium and myocardium by both classical and real-time RT-PCR [11]. In addition, immunocytochemistry evidences indicated that ghrelin can be synthesized by human vascular endothelial cells [19].

The best investigated ghrelin receptor, GHS-R1a, is found on the surface of isolated human cardiomyocytes and the HL-1 cell line [18]. The human myocardium and aorta also extensively express GHS-R1a [11], although to a much lower degree than the pituitary. GHS-R1b mRNA, another transcript from the identical gene of GHS-R1a, is likewise highly expressed in human myocardium [11], but the physiological function of this receptor subtype is yet to be determined. An unknown subtype of ghrelin receptor, distinct from GHS-R1a and GHS-R1b, is found in H9C2 cardiomyocytes and endothelial cells, and this particular ghrelin receptor has the same binding affinity to either acylated or non-acylated ghrelin [20]. In addition, CD36, a type B scavenger receptor of a 84 kD glycoprotein, is expressed in rat and human cardiovascular tissues [21-23] ,and can covalently bind to synthetic peptidyl GHSs, such as [^125^I]Tyr-Ala-hexarelin. However, the potential relationship between ghrelin and CD36 requires further studies. Using a [^125^I]His9-ghrelin binding assay, the pervasiveness of GHS receptors in the cardiovascular system has been confirmed in the human saphenous veins, aorta, coronary artery, left ventricle, and right atrium [24]. Different subtypes of ghrelin receptors vary in their biological effects on heart and blood vessels.

## CARDIOVASCULAR EFFECTS OF GHRELIN

### 1 Vascular Effects of Ghrelin

In healthy volunteers, single intravenous bolus of human ghrelin (10 µg/kg) significantly decreased the mean arterial pressure (MAP) [25]. Ghrelin level has been observed to positively correlate with micro vascular flow rate [26]. The ghrelin’s effect on blood vessels is closely related to nitric oxide (NO). Ghrelin stimulates NO production in bovine aortic endothelium in a time and dose dependent fashion [27]. In human aortic endothelial cells, ghrelin promotes NO synthesis by GHSR-1a, phosphoinositide-3 (PI3K), Akt, and endothelial NO synthetase (eNOS) pathways [27]. In GH-deficient rats, ghrelin enhances the maximal relaxation of the aortic ring and expression of the eNOS in the aorta, and this effect can be inhibited by non-selective NOS inhibitors [28].

However, in cultured human umbilical vein endothelial cells (HUVEC), ghrelin may modulate the vascular tone by negative regulation of mRNA expression of prostaglandin-endoperoxide synthase-2 (COX2), eNOS, and inducible NO synthetase (iNOS) [29]. Another report suggested that ghrelin exerts its contractile activity on guinea-pig renal arteries by facilitation of endothelin-1(ET-1) triggered intracellular signaling in smooth muscle cell, including phospholipase C (PLC), Rho-kinase, and intracellular IP3-sensitive Ca^2+^ release, and/or by stimulating the release of an unknown contractile mediator from the endothelium [30]. Given earlier reports showing the direct vasodilatory effects on endothelium exfoliated blood vessels [31] and contractile effects on coronary arteries [32, 33], the vascular effects of this peptide is quite complex and variable.

### 2 Endothelium Protective Effect of Ghrelin

Application of exogenous ghrelin can improve the impaired endothelium function of patients with metabolic syndrome (MS) by increasing NO bioactivity [34]. Ghrelin is also able to suppress high glucose-induced vascular endothelial cells (ECV-304) apoptosis by activating PI3K/Akt ways and reducing reactive oxygen species (ROS) generation [35]. In addition, ghrelin can inhibit inflammatory response of HUVEC [36] and migration of human aortic endothelial cells (HAEC)[37].

### 3 Cardiac Effect of Ghrelin

Current research has shown that there is an association between systemic ghrelin levels and cardiovascular indexes [38]. In healthy volunteers and patients with chronic congestive heart failure (CHF), ghrelin can decrease peripheral vascular resistance, resulting in an increase in cardiac index and stroke volume [25, 39]. Ghrelin also can improve ventricular remodeling [40, 41], decrease cardiac injury induced by ischemia/reperfusion (I/R) [42] and isoprenaline [43] and reduce infarct size [44]. *In vitro*, ghrelin decreases inotropism [45, 46] and lusitropism [46], protects cardiomyocytes from apoptpsis [20]. In addition, GH is indispensable for the maintenance of heart structure and function, so, ghrelin also has an indirect cardio-protective effect.

## GHRELIN AND CARDIOVASCULAR DISEASES

### 1 Ghrelin and Coronary Atherosclerotic Heart Disease (CAD)

Single nucleotide polymorphisms (SNPs) analyses in human suggests that specific haplotypes of the ghrelin ligand and its receptor act epistatically to affect susceptibility or tolerance to myocardial infarction (MI) and/or CAD [47]. Among CAD patients, those with variant genotypes (Leu72Met and Met72Met) had lower body mass index (BMI) than Leu72Leu carriers [48], however, the preproghrelin Leu72Met polymorphism is not associated with CAD in the Chinese population. Studies involving ghrelin’s role in CAD fall under three major categories:

#### a Regulation of Atherosclerosis 

One study showed that the plasma concentration of ghrelin had a positive correlation with development of carotid artery atherosclerosis in males, but not in females [49]. Animal studies suggest that ghrelin receptors were significantly up-regulated (3-4 fold) in both atherosclerotic coronary arteries and saphenous vein grafts with advanced intimal thickening, when compared with normal vessels [50, 51]. However, a research on kidney transplant patients demonstrates that lower plasma ghrelin concentration is an independent marker for abnormalities in glucose homeostasis, which is related to greater carotid intima-media thickness (cIMT) [52], a well-established surrogate marker for atherosclerosis. Furthermore, findings in older subjects with MS demonstrate that cIMT is significantly inversely correlated with ghrelin levels [53] and in elderly hypertensives des-acyl ghrelin had a significant inverse correlation with cIMT [54]. On the whole, ghrelin and its receptors participate in the occurrence and development of the atherosclerotic process, and elevations of both levels may represent a compensatory mechanism to reverse the process, while, in kidney transplant patients and older people with MS or hypertension, this compensatory ability may be lost or damaged, of course, this deduction need further confirmation.

So far, the potential regulating mechanism of ghrelin on atherosclerosis is not clear. Endothelial dysfunction is considered to be one of the earliest events of the atherosclerotic development. In patients with endothelial dysfunction, plasma ghrelin level decreases [55]; conversely, the application of exogenous ghrelin can improve endothelial dysfunction in MS patients by increasing nitric oxide bioactivity [34]. In addition, atherosclerosis is linked to inflammation and immunological reaction. Studies found that, ghrelin can inhibit proinflammatory cytokine pro-duction, mononuclear cell binding, and nuclear factor-kappa B(NF-κB) activation in human endothelial cells in vitro as well as endotoxin-induced cytokine production in vivo [36], moreover, exogenous ghrelin may significantly inhibit TNF-α/interferon-γ-induced CD40 expression in HUVEC cells in a concentration-dependent manner [56]. These novel antiinflammatory and immunoregulatory actions of ghrelin may play a further modulatory role in atherosclerosis. In addition, in obese subjects low circulating levels of active ghrelin may enhance oxidative stress and the process of atherosclerosis [57], hence, ghrelin, through its anti-oxidative effects, may attenuate atherosclerosis. However, ghrelin in pathophysiologic concentrations, as seen in patients with anorexia nervosa, Prader-Willi syndrome, and type 2 diabetes, increases endothelial cell adhesion molecule (intercellular adhesion molecule-1, ICAM-1; vascular cell adhesion molecule-1,VCAM-1) expression, possibly contributing to increased atherosclerosis risk in such subjects [58]. Therefore, elucidation of the precise mechanism by which ghrelin regulates atherosclerosis may provide key insights into ghrelin’s administration in atherosclerosis therapy.

#### b Protective Effect of Ghrelin on Myocardial I/R Injury

Administration of ghrelin during reperfusion has been demonstrated to protect the myocardium against I/R injury, including reduced myocardial release of lactate dehydrogenase and myoglobin, with subsequent improvements in coronary flow, heart rate, left ventricular systolic pressure, and left ventricular end-diastolic pressure [42]. The cardioprotective effects of ghrelin are independent of growth hormone release and likely involve binding to cardiovascular receptors, a process that is up regulated during I/R [42]. The direct protective effect of ghrelin against I/R is at least partially mediated by inhibiting myocardial endoplasmic reticulum stress (ERS) (data in press).

One study showed that ghrelin can dose-dependently increase coronary perfusion pressure in isolated perfused rat hearts and constrict isolated coronary arterioles, and this coronary vasoconstrictive action is dependent on Ca^2+^ and protein kinase C (PKC) [33]. Another study reported that, in anesthetized pigs, intracoronary infusion of ghrelin induced coronary artery contraction, thereby reducing coronary circulation and the mechanism of this response has been shown to involve the inhibition of beta_2_-adrenergic mediated release of NO [32]. Thus, the exact ghrelin effects on coronary perfusion are inconclusive at present. Nonetheless, the beneficial effects of ghrelin on isolated I/R heart may yet indicate a potential role for this peptide in prevention of I/R injury.

#### c Potential Benefits in MI

Plasma C-ghrelin (66 carboxyl-terminal amino acids of pro-ghrelin) and ghrelin levels significantly decrease after MI [59]. While, ghrelin administration significantly reduced infarct size in isolated working rat hearts; the cardio-protective effect is independent of growth hormone and may be related to PKC activation [44]. Moreover, in post-MI rats, acute administration of ghrelin (100 µg/kg sc, twice daily, for 2 wk), compared with saline controls, left ventricle(LV)end-diastolic pressure and LV enlargement were substantially less; the peak rate of rise and fall of LV pressure significantly increased; the increase in morphometrical collagen volume fraction in the non-infarct regions were attenuated, accompanied by suppression of collagen I and III mRNA levels; the MI-induced increases in heart rate and plasma norepinephrine concentration were dramatically suppressed; and lastly, in this model, the ratio of low-to-high frequency spectra of heart rate variability was likewise attenuated [60]. Thus, future clinical trials are needed to reveal the potential role of ghrelin in improvement of post-MI prognosis.

### 2 Ghrelin and Hypertension

Some recent data have implicated reduced plasma ghrelin levels in patients with hypertension [61]. Low-level of ghrelin has been independently associated with elevated blood pressure (BP) [61]. The ghrelin Arg51Gln mutation, which is associated with low plasma ghrelin concentration, may be a risk factor for development of hypertension [62]. Moreover, SNPs analyses in subjects with impaired glucose tolerance has demonstrated the association of several ghrelin gene variations with BP levels [63]. However, in obese women, ghrelin was positively associated with hypertension; and this association was inversely influenced by the increase of BMI [64]. Therefore, the exact relationship between obesity, hypertension and ghrelin awaits further elucidation. Pregnant, spontaneously hypertensive rats (SHR) exhibit significantly higher plasma ghrelin levels when compare with those of pregnant or normotensive Wistar-Kyoto (WKY) animals. In contradistinction, the mRNA of ghrelin in the placenta of SHR was lower than that of normotensive rats, suggesting a possible association of ghrelin and hypertension during pregnancy [65]. Makino and colleagues investigated the ghrelin concentrations in plasma of non-pregnant women, normal pregnant women, patients with pregnancy-induced hypertension, and postpartum women and they reported a negative correlation between plasma ghrelin concentration and systemic BP in normal pregnant women. Conversely, women with pregnancy-induced hypertension have significantly higher levels of ghrelin than those of normal pregnant women, and there is a significant correlation between plasma ghrelin levels and systemic BP [66]. These data suggest that the potential relationship between ghrelin and hypertension in women may differ depending on the gravid state.

As a potent vasodilator, ghrelin can decrease BP. Exogenous administration of both ghrelin and non-acylghrelin revealed endothelium-independent angioectatic potency and efficacy in reversing ET-1 induced constriction in human isolated arteries [19]. Furthermore, intravenous ghrelin injection has been shown to significantly decrease MAP in humans [25], suggesting a potential therapeutic role in hypertension.

The exact BP regulatory mechanism by ghrelin is unknown but may involve central and peripheral pathways.  In the brain, ghrelin has been demonstrated to suppress sympathetic activity and decrease BP [67]. Additionally, ghrelin, when incubated with isolated SHR aortas, can suppress vascular oxidative stress, thereby improve hypertension [68]. Modulation of endothelial function, as discussed previously, may be at least partially responsible for the anti-hypertensive actions of ghrelin. In addition, another study suggested that the calcium-activated potassium-channel may play a key role in ghrelin-evoked decreases in MAP, especially in situations of endothelial dysfunction associated with paucity of nitric oxide [69].

### 3 Ghrelin and Cardiomyopathy 

Early studies focused on the protective effect of recombinant GH and insulin-like growth factor-1 (IGF-1) in cardiomyopathy [70], including ischemic cardiomyopathy, dilated cardiomyopathy (DCM), and tachycardia induced cardiomyopathy. In recent years, ghrelin and GHS are studied in this field. Chronic subcutaneous administration of ghrelin (100μg/kg, bid, three weeks) can improve LV dysfunction and attenuate the development of LV remodeling [40]. Administration of synthetic GSH, such as hexarelin and GHRP-6, have also demonstrated improvements in patients and animals with ischemic cardiomyopathy or dialated cardiomyopathy (DCM), independent of IGF-1 [71-75]. However, the role of ghrelin in the treatment of acromegaly-induced cardiomyopathy remains controversial [76].

At present, the therapeutic effects of ghrelin on cardiomyopathy are postulated to involve its inhibition of apoptosis of cardiomyocytes, by both GH-IGF-1-dependent [77] and–independent [20, 78, 79] pathways. Although with optimal therapy with angiotensin-converting enzyme inhibitors, diuretics, digoxin, and beta-adrenergic receptor blockers, the average survival for cardiomyopathy sufferers with CHF is only three to five years [80]. Further work may reveal ghrelin to be a promising addition in our armamentarium against this formidable condition.

### 4 Ghrelin and Drug-Induced Myocardial Injury

In rat models of isoproterenol (ISO) [43] –or adriamycin (ADR) [81] –induced myocardial injury, endogenous plasma ghrelin levels increased significantly, perhaps due to a compensatory, self-protective mechanism. Moreover, administration of exogenous ghrelin can protect against ISO-induced myocardial injury [43]; and in *in vitro* studies, ghrelin has shown to prevent against the cardio-toxicity of ADR [82]. The anti-ISO myocardial injury effects of ghrelin may be related to its inhibition of ET-1 [43], while its protective effects against ADR may be attributable to upregulation of TNF-α/NF-κB pathways as well as its  mitochondrial stabilization properties [82]. Thus far, however, definitive data regarding therapeutic benefits in drug-induced cardiomyopathies remain lacking.

### 5 Ghrelin in Congenital Heart Diseases and Pulmonary Hypertension (PH)

One study reported that, in patients with congenital heart diseases, serum ghrelin levels are significantly higher than those of controls and correlated with TNF-α levels [83]. The increased ghrelin levels may be related to malnutrition and growth retardation, and the correlation of ghrelin and cytokines may be associated with CHF and chronic shunt-induced hypoxemia [83]. Whether ghrelin can affect formation of the heart tube and development of the embryo heart is not known.  In chronically hypoxic rats, daily subcutaneous ghrelin injection (150 μg/kg) for two weeks  can significantly attenuate the development of PH, pulmonary vascular remodeling, right ventricular (RV) hypertrophy, and overexpression of eNOs and ET-1, as compared with saline control group [84]. In a rat model of monocrotaline (MCT)-induced PH, endogenous ghrelin expression increased in RV myocardium, while exogenous administration of ghrelin attenuated PH, RV hypertrophy, peripheral pulmonary arterial wall thickness, RV diastolic impairment, and LV dysfunction [41].

### 6 Ghrelin and Peripheral Vascular Disease

Ghrelin has been observed to retard vascular calcification. The aortic calcification induced by vitamin D3 and nicotine, as well as VSMC calcification induced by beta-glycerophosphate, are significantly attenuated by ghrelin [85]. In addition, ghrelin inhibits the angiotensin-II induced HAEC migration by increasing intracellular concentration of cAMP [37], which may further be involved in the prevention of the vascular calcification process. Ghrelin has been reported to suppress high glucose-induced vascular endothelial cell (ECV-304) apoptosis by activating PI3K/Akt pathways and reducing ROS generation [35]. This effect of ghrelin may be useful in the prevention of diabetic vascular complications, especially in obese patients. In addition, as mentioned above, ghrelin is also involved in the regulation of peripheral atherosclerosis.

### 7 Ghrelin and Heart Failure (HF)

Studies have suggested multi-protective effects of ghrelin on heart failure.

#### a Improvement in Cardiac Function 

Animal experiments show that ghrelin can evoke significant decrease in MAP in normal, CHF, and GH deficient rats [40], and similar effects is observed in humans: in healthy volunteers and patients with CHF, ghrelin can decrease vascular resistance, increase cardiac index and stroke volume [25, 39]. This action is related to its direct vasodilatory effect, as well as inhibition of the sympathetic activity [86]. While, Wiley and co-investigators outlined its endothelium-independent vasodilative properties and its antagonistic action against ET-1 [31]. And correlation has been observed between ghrelin levels and cardiac index [38], and Enomoto *et al*. reported that ghrelin can dose-dependently increase stroke volume, cardiac index, and LVd*p*/d*t*_max_ in the absence of significant changes in heart rate [87]. However, neither ghrelin nor non- acyl-ghrelin has demonstrated effects on contractile force in paced atria [19], and *in vitro* studies failed to show a direct increase in contractility of cardiomyocytes [88], thus, ghrelin-associated improvements in cardiac function may be attributed to afterload reduction and its stimulation of secretion of GH, a myocardial stimulant.

In addition, ghrelin has been noted to exert suppressive effects on  papillary muscle contraction and relaxation in rats with MCT-induced right ventricular hypertrophy [88]. Moreover, in both normal and hypertrophied myocardial cells, ghrelin inhibits contraction, relaxation and premature relaxation [46], so ghrelin may also improve myocardial function by reducing myocardial oxygen consumption.

#### b Anti-Cardiac Cachexia Effect of Ghrelin

Prevention, or at least postponement, of the process of cachexia is a basic strategy in the treatment of heart failure [89]. Although non-cachectic CHF patients demonstrate a normal ghrelin level, plasma ghrelin is significantly higher in CHF patients with cachexia (heart failure for more than six months, a non-edemetous and unintentional weight loss over 6% of the baseline weight) [90]. Cardiac cachexia, characterized by weight loss and muscle consumption, is often observed in end-stage CHF and is a strong independent risk factor for mortality in these individuals. In CHF rats treated with ghrelin, appropriate weight gain and muscle/bone ratio can be maintained [40]. The mechanism may be attributed to the following ghrelin properties: appetite stimulation; enhanced fat and carbohydrate metabolism, resulting in positive energy balance; increased secretion of GH and IGF-1, both of which can promote anabolic proliferation of skeletal and cardiac muscle. Ghrelin exerts a generalized anti-catabolic effect in a variety of diseases. Aside from its classic regulation of appetite and fat metabolism, ghrelin also plays an important role in the regulation of fat-free mass [91].

#### c Inhibition of Myocardial Apoptosis and Improvement of Ventricular Remodeling

It is also observed that in pressure overload rats with heart failure the application of synthetic peptide GHS (GHRP-1, 2, 6, hexarelin) can protect myocardial cells against apoptosis, with the increase of GHS-R1a [92]. Furthermore, *in vitro* data suggest that hexarelin can inhibit angiotensin-II (AngII)-induced myocardial apoptosis [79]. On these grounds, we speculate that ghrelin may also play anti-myocardial apoptosis role through the GHS-R1a. In addition, Bedendi, and coauthors reported, the acyl-ghrelin and non- acyl-ghrelin prevented cardiac myocyte apoptosis through the activation of ERK-1/2 and Akt [20], perhaps mediated through a new receptor distinct from 1a and 1b subtypes.

Early studies have shown that both GH and IGF-1 are necessary for cardiac and skeletal muscle growth as well as energy homeostasis [93, 94]. While, in patients with GH-deficiency, there are deficiencies in cardiac muscle mass and diastolic filling. Furthermore, addition of GH may be beneficial to the CHF patients' cardiac structure and function [95-97]. Experiments have shown that, in CHF rats, ghrelin administration (100μg/kg, bid, 3 weeks) can increase plasma IGF-1 and long-term administration of ghrelin can increase ventricular posterior wall thickness, delay progression of LV dilatation, and decrease wall stress [40]. So it is postulated that ghrelin may inhibit ventricular remodeling through the GH-IGF-1. Intravenous ghrelin administration to CHF patients for 3 weeks has been shown to increase LV ejection fraction (LVEF), LV mass, [40] maximal exercise tolerance, and maximal oxygen consumption, while decrease LV end-diastolic volume [98].

Ghrelin can improve ventricular remodeling after MI [60] and reduce the diameter of cardiac muscle fibers in RV hypertrophy induced by PH [99]. Fibroblast proliferation and collagen synthesis, integral components in the detrimental process of myocardial remodeling, are inhibited *in vitro* by hexarelin in rat cardiomyocytes [100]. Given the possible common receptor of the two molecules, ghrelin may also have comparable properties. Hence, we have reason to believe that, ghrelin improve ventricular remodeling by GH-dependent and independent pathways.

#### d Anti-Neuroendocrine Effect of Ghrelin

Ghrelin has been shown to exert direct suppression of central nervous system sympathetic output [86, 101] and the concentration of norepinephrine in patients with CHF [98].  In pressure overload rats, the peptide GHS (GHRP-1, 2,6, hexarelin) can significantly inhibit CHF-induced increase in catecholamine, renin-angiotensin II, aldosterone, ET-1 and atrial natriuretic polypeptide [92]. So, we deduce that ghrelin may also have the similar effects.

#### e Anti-Inflammatory and Anti-Oxidative Effects

CHF is a complex syndrome, the pathophysiology of which involves neuroendocrine activation, heightened inflammatory response, and increased oxidative stress [102]. Experiments have proved that, ghrelin, both* in vivo* and *in vitro* has anti-inflammatory effect [36]. In addition, extensive data have confirmed ghrelin’s role in the antioxidation. Ghrelin pre-incubation of aortic segments from SHR has demonstrated attenuation in vascular superoxide production and NAD(P)H oxidase activity [68]. Ghrelin reperfusion could decrease the content of lipid peroxidation product malondialdehyde in myocardium of I/R in rat hearts [42]. Moreover, ghrelin’s anti-oxidative effect has also been confirmed in studies in digestive [103, 104], nervous [105] and endocrine [106] systems. Therefore, these functions may be responsible for the beneficial action of ghrelin in heart failure.

### 8 Ghrelin and the Metabolic Risk Factors of Cardiovascular System 

It is well known that type 2 diabetes and MS are risk factors of many cardiovascular diseases. In humans acyl-ghrelin reduces insulin sensitivity while non-acyl-ghrelin has opposite effects and ghrelin seems to have diabetogenic effects [107]. Low plasma concentrations of ghrelin are associated with several components of the MS, such as obesity and insulin resistance, however, ghrelin infusion acutely induces lipolysis and insulin resistance in human [108]. Hence, it seems that ghrelin has no good effect on these cardiovascular risk factors like MS and the cardiovascular benefit of people with these risk factors needs further studies.

## CARDIOVASCULAR PROTECTIVE EFFECTS AND PATHWAYS

According to the available data, mechanisms by which ghrelin carries out its cardiovascular protective effects are relatively complicated, and can be classified as GH dependent pathway and GH independent pathway. The cardiovascular protective effects and the according mechanisms are summarized in Table **1**.

## CONCLUSION

In summary, there is robust data supporting an association between ghrelin and various cardiovascular conditions, and some common processes such as inflammation, oxidative stress, and ERS have been implicated, although the exact mechanisms have not been fully elucidated. Preliminary studies have suggested this novel peptide has a promising prospect. To date, no adverse effect of this peptide has been reported, although further investigations are needed to define any potential therapeutic roles. In future, to approach the in-depth mechanisms of ghrelin in cardiovascular homeostasis regulation will be the investigative focal point.

## Figures and Tables

**Table 1:** Ghrelin’s Cardiovascular Protective Effects and According Mechanisms

Disease	Protective Effect	Mechanism	
CAD	Regulation of atherosclerosis	*GH independent pathway:*	1) improve endothelial dysfunction
			2) antiinflammatory action
			3) immunoregulatory action
	Protective effect of ghrelin on myocardial I/R injury	*GH independent pathway:*	1) inhibit myocardial endoplasmic reticulum stress
			2) increase coronary perfusion?
			3) anti-oxidative effects
			4) improve myocardial metabalism?
	Potential benefits in MI	*GH independent pathway:*	1) inhibit myocardial apoptosis (probablely)
			2) inhibit myocardial fibrosis (probablely)
hypertension		*GH independent pathway:*	1) vasodilation
			2) suppress vascular oxidative stress
			3) modulate of endothelial function
			4) suppress sympathetic nervous system
cardiomyopathy		*GH dependent pathway*	inhibit myocardial apoptosis
		*GH independent pathway:*	1) inhibit myocardial apoptosis (maybe)
			2) improve ventricular remodeling
drug-induced myocardial injury		*GH independent pathway*	1) inhibit myocardial apoptosis
			2) anti-oxidative effect
			3) Anti-inflammatory effect
pulmonary hypertension		*GH independent pathway:*	1) attenuate pulmonary arterial hypertension
			2) decrease RV hypertrophy
peripheral vascular disease		*GH independent pathway:*	retard vascular calcification
			regulation of peripheral atherosclerosis.
heart failure	Improvement in cardiac function	*GH independent pathway:*	1) vasodilation
			2) reducing myocardial oxygen consumption
	Anti-cardiac cachexia effect of ghrelin	*GH independent pathway:*	1) appetite stimulation;
			2) enhance fat and carbohydrate metabolism
			3) antiinflammatory action
			4) anti-oxidative effects
		*GH dependent pathway*	promote anabolic proliferation of skeletal and cardiac muscle
	Improvement of ventricular remodeling	*GH independent pathway:*	1) anti-myocardial apoptosis
			2) inhibit myocardial fibrosis
		*GH dependent pathway*	1) promote cardiac and skeletal muscle growth
			2) anti-myocardial apoptosis
	Anti-neuroendocrine effect of ghrelin	*GH independent pathway:*	suppress sympathetic nervous system

## References

[1] Kojima M, Hosoda H, Date Y (1999). Ghrelin is a growth-hormone-releasing acylated peptide from stomach. Nature.

[2] Kojima M, Kangawa K (2005). Ghrelin: structure and function. Physiol Rev.

[3] Yang J, Brown MS, Liang G, Grishin NV, Goldstein JL (2008). Identification of the acyltransferase that octanoylates ghrelin, an appetite-stimulating peptide hormone. Cell.

[4] Date Y, Kojima M, Hosoda H (2000). Ghrelin, a novel growth hormone-releasing acylated peptide, is synthesized in a distinct endocrine cell type in the gastrointestinal tracts of rats and humans. Endocrinology.

[5] van der Lely AJ, Tschop M, Heiman ML, Ghigo E (2004). Biological, physiological, pathophysiological, and pharmacological aspects of ghrelin. Endocr Rev.

[6] Hosoda H, Kojima M, Kangawa K (2006). Biological, physiological, and pharmacological aspects of ghrelin. J Pharmacol Sci.

[7] Kojima M, Kangawa K (2006). Drug insight: The functions of ghrelin and its potential as a multitherapeutic hormone. Nat Clin Pract Endocrinol Metab.

[8] Davenport AP, Bonner TI, Foord SM (2005). International Union of Pharmacology. LVI. Ghrelin receptor nomenclature, distribution, and function. Pharmacol Rev.

[9] Tritos NA, Kokkotou EG (2006). The physiology and potential clinical applications of ghrelin, a novel peptide hormone. Mayo Clin Proc.

[10] Rocha-Sousa A, Saraiva J, Henriques-Coelho T (2006). Ghrelin as a novel locally produced relaxing peptide of the iris sphincter and dilator muscles. Exp Eye Res.

[11] Gnanapavan S, Kola B, Bustin SA (2002). The tissue distribution of the mRNA of ghrelin and subtypes of its receptor, GHS-R, in humans. J Clin Endocrinol Metab.

[12] Moller N, Nygren J, Hansen TK (2003). Splanchnic release of ghrelin in humans. J Clin Endocrinol Metab.

[13] Popovic V, Svetel M, Djurovic M (2004). Circulating and cerebrospinal fluid ghrelin and leptin: potential role in altered body weight in Huntington's disease. Eur J Endocrinol.

[14] Kamegai J, Tamura H, Shimizu T (2004). The role of pituitary ghrelin in growth hormone (GH) secretion: GH-releasing hormone-dependent regulation of pituitary ghrelin gene expression and peptide content. Endocrinology.

[15] Kojima M, Kangawa K (2009). Structure and Function of Ghrelin. Results Probl Cell Differ.

[16] Leite-Moreira AF, Soares JB (2007). Physiological, pathological and potential therapeutic roles of ghrelin. Drug Discov Today.

[17] Sun Y, Ahmed S, Smith RG (2003). Deletion of ghrelin impairs neither growth nor appetite. Mol Cell Biol.

[18] Iglesias MJ, Pineiro R, Blanco M (2004). Growth hormone releasing peptide (ghrelin) is synthesized and secreted by cardiomyocytes. Cardiovasc Res.

[19] Kleinz MJ, Maguire JJ, Skepper JN, Davenport AP (2006). Functional and immunocytochemical evidence for a role of ghrelin and desoctanoyl ghrelin in the regulation of vascular tone in man. Cardiovasc Res.

[20] Baldanzi G, Filigheddu N, Cutrupi S (2002). Ghrelin and des-acyl ghrelin inhibit cell death in cardiomyocytes and endothelial cells through ERK1/2 and PI 3-kinase/AKT. J Cell Biol.

[21] Bodart V, Bouchard JF, McNicoll N (1999). Identification and characterization of a new growth hormone-releasing peptide receptor in the heart. Circ Res.

[22] Muccioli G, Broglio F, Valetto MR (2000). Growth hormone-releasing peptides and the cardiovascular system. Ann Endocrinol.

[23] Papotti M, Ghe C, Cassoni P (2000). Growth hormone secretagogue binding sites in peripheral human tissues. J Clin Endocrinol Metab.

[24] Cao JM, Ong H, Chen C (2006). Effects of ghrelin and synthetic GH secretagogues on the cardiovascular system. Trends Endocrinol Metab.

[25] Nagaya N, Kojima M, Uematsu M (2001). Hemodynamic and hormonal effects of human ghrelin in healthy volunteers. Am J Physiol Regul Integr Comp Physiol.

[26] Tigno XT, Selaru IK, Angeloni SV, Hansen BC (2003). Is microvascular flow rate related to ghrelin, leptin and adiponectin levels?. Clin Hemorheol Microcirc.

[27] Iantorno M, Chen H, Kim JA (2007). Ghrelin has novel vascular actions that mimic PI 3-kinase-dependent actions of insulin to stimulate production of NO from endothelial cells. Am J Physiol Endocrinol Metab.

[28] Shimizu Y, Nagaya N, Teranishi Y (2003). Ghrelin improves endothelial dysfunction through growth hormone-independent mechanisms in rats. Biochem Biophys Res Commun.

[29] Minici F, Miceli F, Tiberi F (2007). Ghrelin in vitro modulates vasoactive factors in human umbilical vein endothelial cells. Fertil Steril.

[30] Dimitrova DZ, Mihov DN, Wang R (2007). Contractile effect of ghrelin on isolated guinea-pig renal arteries. Vasc Pharmacol.

[31] Wiley KE, Davenport AP (2002). Comparison of vasodilators in human internal mammary artery: ghrelin is a potent physiological antagonist of endothelin-1. Br J Pharmacol.

[32] Grossini E, Molinari C, Mary DA (2007). Intracoronary ghrelin infusion decreases coronary blood flow in anesthetized pigs. Endocrinology.

[33] Pemberton CJ, Tokola H, Bagi Z (2004). Ghrelin induces vasoconstriction in the rat coronary vasculature without altering cardiac peptide secretion. Am J Physiol Heart Circ Physiol.

[34] Tesauro M, Schinzari F, Iantorno M (2005). Ghrelin improves endothelial function in patients with metabolic syndrome. Circulation.

[35] Zhao H, Liu G, Wang Q (2007). Effect of ghrelin on human endothelial cells apoptosis induced by high glucose. Biochem Biophys Res Commun.

[36] Li WG, Gavrila D, Liu X (2004). Ghrelin inhibits proinflammatory responses and nuclear factor-kappaB activation in human endothelial cells. Circulation.

[37] Rossi F, Bertone C, Petricca S, Santiemma V (2007). Ghrelin inhibits angiotensin II-induced migration of human aortic endothelial cells. Atherosclerosis.

[38] Tritos NA, Kissinger KV, Manning WJ, Danias PG (2004). Association between ghrelin and cardiovascular indexes in healthy obese and lean men. Clin Endocrinol (Oxf).

[39] Nagaya N, Miyatake K, Uematsu M (2001). Hemodynamic, renal, and hormonal effects of ghrelin infusion in patients with chronic heart failure. J Clin Endocrinol Metab.

[40] Nagaya N, Uematsu M, Kojima M (2001). Chronic administration of ghrelin improves left ventricular dysfunction and attenuates development of cardiac cachexia in rats with heart failure. Circulation.

[41] Henriques-Coelho T, Correia-Pinto J, Roncon-Albuquerque R (2004). Endogenous production of ghrelin and beneficial effects of its exogenous administration in monocrotaline-induced pulmonary hypertension. Am J Physiol Heart Circ Physiol.

[42] Chang L, Ren Y, Liu X (2004). Protective effects of ghrelin on ischemia/reperfusion injury in the isolated rat heart. J Cardiovasc Pharmacol.

[43] Chang L, Zhao J, Li GZ (2004). Ghrelin protects myocardium from isoproterenol-induced injury in rats. Acta Pharmacol Sin.

[44] Frascarelli S, Ghelardoni S, Ronca-Testoni S, Zucchi R (2003). Effect of ghrelin and synthetic growth hormone secretagogues in normal and ischemic rat heart. Basic Res Cardiol.

[45] Bedendi I, Alloatti G, Marcantoni A (2003). Cardiac effects of ghrelin and its endogenous derivatives des-octanoyl ghrelin and des-Gln14-ghrelin. Eur J Pharmacol.

[46] Soares JB, Rocha-Sousa A, Castro-Chaves P, Henriques-Coelho T, Leite-Moreira AF (2006). Inotropic and lusitropic effects of ghrelin and their modulation by the endocardial endothelium, NO, prostaglandins, GHS-R1a and KCa channels. Peptides.

[47] Baessler A, Fischer M, Mayer B (2007). Epistatic interaction between haplotypes of the ghrelin ligand and receptor genes influence susceptibility to myocardial infarction and coronary artery disease. Hum Mol Genet.

[48] Tang NP, Wang LS, Yang L (2008). Preproghrelin Leu72Met polymorphism in Chinese subjects with coronary artery disease and controls. Clin Chim Acta.

[49] Poykko SM, Kellokoski E, Ukkola O (2006). Plasma ghrelin concentrations are positively associated with carotid artery atherosclerosis in males. J Intern Med.

[50] Katugampola SD, Maguire JJ, Kuc RE, Wiley KE, Davenport AP (2002). Discovery of recently adopted orphan receptors for apelin, urotensin II, and ghrelin identified using novel radioligands and functional role in the human cardiovascular system. Can J Physiol Pharmacol.

[51] Katugampola SD, Pallikaros Z, Davenport AP (2001). [125I-His(9)]-ghrelin, a novel radioligand for localizing GHS orphan receptors in human and rat tissue: up-regulation of receptors with athersclerosis. Br J Pharmacol.

[52] Genis BB, Granada ML, Alonso N (2007). Ghrelin, glucose homeostasis, and carotid intima media thickness in kidney transplantation. Transplantation.

[53] Kotani K, Sakane N, Saiga K (2006). Serum ghrelin and carotid atherosclerosis in older Japanese people with metabolic syndrome. Arch Med Res.

[54] Yano Y, Toshinai K, Inokuchi T (2009). Plasma des-acyl ghrelin, but not plasma HMW adiponectin, is a useful cardiometabolic marker for predicting atherosclerosis in elderly hypertensive patients. Atherosclerosis.

[55] Tajtakova M, Petrovicova J, Spurny P (2005). Selected hormones levels in individuals with endothelial dysfunction and insulin resistance. Bratisl Lek Listy.

[56] Zhang M, Yuan F, Chen H, Qiu X, Fang W (2007). Effect of exogenous ghrelin on cell differentiation antigen 40 expression in endothelial cells. Acta Biochim Biophys Sin (Shanghai).

[57] Suematsu M, Katsuki A, Sumida Y (2005). Decreased circulating levels of active ghrelin are associated with increased oxidative stress in obese subjects. Eur J Endocrinol.

[58] Skilton MR, Nakhla S, Sieveking DP, Caterson ID, Celermajer DS (2005). Pathophysiological levels of the obesity related peptides resistin and ghrelin increase adhesion molecule expression on human vascular endothelial cells. Clin Exp Pharmacol Physiol.

[59] Pemberton C, Wimalasena P, Yandle T, Soule S, Richards M (2003). C-terminal pro-ghrelin peptides are present in the human circulation. Biochem Biophys Res Commun.

[60] Soeki T, Kishimoto I, Schwenke DO (2008). Ghrelin suppresses cardiac sympathetic activity and prevents early left ventricular remodeling in rats with myocardial infarction. Am J Physiol Heart Circ Physiol.

[61] Poykko SM, Kellokoski E, Horkko S (2003). Low plasma ghrelin is associated with insulin resistance, hypertension, and the prevalence of type 2 diabetes. Diabetes.

[62] Poykko S, Ukkola O, Kauma H, Savolainen MJ, Kesaniemi YA (2003). Ghrelin Arg51Gln mutation is a risk factor for Type 2 diabetes and hypertension in a random sample of middle-aged subjects. Diabetologia.

[63] Mager U, Kolehmainen M, Lindstrom J (2006). Association between ghrelin gene variations and blood pressure in subjects with impaired glucose tolerance. Am J Hypertens.

[64] Oner-Iyidogan Y, Kocak H, Gurdol F (2007). Circulating ghrelin levels in obese women: a possible association with hypertension. Scand J Clin Lab Invest.

[65] Raso GM, Bianco G, Iacono A (2007). Maternal adaptations to pregnancy in spontaneously hypertensive rats: leptin and ghrelin evaluation. J Endocrinol.

[66] Makino Y, Hosoda H, Shibata K (2002). Alteration of plasma ghrelin levels associated with the blood pressure in pregnancy. Hypertension.

[67] Matsumura K, Tsuchihashi T, Fujii K, Iida M (2003). Neural regulation of blood pressure by leptin and the related peptides. Regul Pept.

[68] Kawczynska-Drozdz A, Olszanecki R, Jawien J (2006). Ghrelin inhibits vascular superoxide production in spontaneously hypertensive rats. Am J Hypertens.

[69] Shinde UA, Desai KM, Yu C, Gopalakrishnan V (2005). Nitric oxide synthase inhibition exaggerates the hypotensive response to ghrelin: role of calcium-activated potassium channels. J Hypertens.

[70] Marleau S, Mulumba M, Lamontagne D, Ong H (2006). Cardiac and peripheral actions of growth hormone and its releasing peptides: relevance for the treatment of cardiomyopathies. Cardiovasc Res.

[71] Tivesten A, Bollano E, Caidahl K (2000). The growth hormone secretagogue hexarelin improves cardiac function in rats after experimental myocardial infarction. Endocrinology.

[72] De Gennaro Colonna V, Rossoni G, Bernareggi M, Muller EE, Berti F (1997). Cardiac ischemia and impairment of vascular endothelium function in hearts from growth hormone-deficient rats: protection by hexarelin. Eur J Pharmacol.

[73] Iwase M, Kanazawa H, Kato Y (2004). Growth hormone-releasing peptide can improve left ventricular dysfunction and attenuate dilation in dilated cardiomyopathic hamsters. Cardiovasc Res.

[74] Shen YT, Lynch JJ, Hargreaves RJ, Gould RJ (2003). A growth hormone secretagogue prevents ischemic-induced mortality independently of the growth hormone pathway in dogs with chronic dilated cardiomyopathy. J Pharmacol Exp Ther.

[75] Imazio M, Bobbio M, Broglio F (2002). GH-independent cardiotropic activities of hexarelin in patients with severe left ventricular dysfunction due to dilated and ischemic cardiomyopathy. Eur J Heart Fail.

[76] Schwarz ER, Jammula P, Gupta R, Rosanio S (2006). A case and review of acromegaly-induced cardiomyopathy and the relationship between growth hormone and heart failure: cause or cure or neither or both?. J Cardiovasc Pharmacol Ther.

[77] Wang L, Ma W, Markovich R, Chen JW, Wang PH (1998). Regulation of cardiomyocyte apoptotic signaling by insulin-like growth factor I. Circ Res.

[78] Filigheddu N, Fubini A, Baldanzi G (2001). Hexarelin protects H9c2 cardiomyocytes from doxorubicin-induced cell death. Endocrine.

[79] Pang JJ, Xu RK, Xu XB (2004). Hexarelin protects rat cardiomyocytes from angiotensin II-induced apoptosis in vitro. Am J Physiol Heart Circ Physiol.

[80] Perrot A, Ranke MB, Dietz R, Osterziel KJ (2001). Growth hormone treatment in dilated cardiomyopathy. J Card Surg.

[81] Xu Z, Wu W, Zhang X, Liu G (2007). Endogenous ghrelin increases in adriamycin-induced heart failure rats. J Endocrinol Invest.

[82] Xu Z, Lin S, Wu W (2008). Ghrelin prevents doxorubicin-induced cardiotoxicity through TNF-alpha/NF-kappaB pathways and mitochondrial protective mechanisms. Toxicology.

[83] Yilmaz E, Ustundag B, Sen Y (2007). The levels of Ghrelin, TNF-alpha, and IL-6 in children with cyanotic and acyanotic congenital heart disease. Mediat Inflamm.

[84] Schwenke DO, Tokudome T, Shirai M (2008). Exogenous ghrelin attenuates the progression of chronic hypoxia-induced pulmonary hypertension in conscious rats. Endocrinology.

[85] Li GZ, Jiang W, Zhao J (2005). Ghrelin blunted vascular calcification *in vivo* and *in vitro* in rats. Regul Pept.

[86] Lin Y, Matsumura K, Fukuhara M (2004). Ghrelin acts at the nucleus of the solitary tract to decrease arterial pressure in rats. Hypertension.

[87] Enomoto M, Nagaya N, Uematsu M (2003). Cardiovascular and hormonal effects of subcutaneous administration of ghrelin, a novel growth hormone-releasing peptide, in healthy humans. Clin Sci (Lond).

[88] Soares JB, Rocha-Sousa A, Castro-Chaves P (2005). Contractile effects of Ghrelin and expression of its receptor GHS-R1a in normal and hypertrophic myocardium. Rev Port Cardiol.

[89] Strassburg S, Anker SD (2006). Metabolic and immunologic derangements in cardiac cachexia: where to from here?. Heart Fail Rev.

[90] Nagaya N, Uematsu M, Kojima M (2001). Elevated circulating level of ghrelin in cachexia associated with chronic heart failure: relationships between ghrelin and anabolic/catabolic factors. Circulation.

[91] Akamizu T, Kangawa K (2007). Emerging results of anticatabolic therapy with ghrelin. Curr Opin Clin Nutr Metab Care.

[92] Xu XB, Pang JJ, Cao JM (2005). GH-releasing peptides improve cardiac dysfunction and cachexia and suppress stress-related hormones and cardiomyocyte apoptosis in rats with heart failure. Am J Physiol Heart Circ Physiol.

[93] Amato G, Carella C, Fazio S (1993). Body composition, bone metabolism, and heart structure and function in growth hormone (GH)-deficient adults before and after GH replacement therapy at low doses. J Clin Endocrinol Metab.

[94] Fuller SJ, Mynett JR, Sugden PH (1992). Stimulation of cardiac protein synthesis by insulin-like growth factors. Biochem J.

[95] Yang R, Bunting S Gillett (1995). Growth hormone improves cardiac performance in experimental heart failure. Circulation.

[96] Cittadini A, Stromer H, Katz SE (1996). Differential cardiac effects of growth hormone and insulin-like growth factor-1 in the rat. A combined in vivo and in vitro evaluation. Circulation.

[97] Fazio S, Sabatini D, Capaldo B (1996). A preliminary study of growth hormone in the treatment of dilated cardiomyopathy. N Engl J Med.

[98] Nagaya N, Moriya J, Yasumura Y (2004). Effects of ghrelin administration on left ventricular function, exercise capacity, and muscle wasting in patients with chronic heart failure. Circulation.

[99] Henriques-Coelho T, Roncon-Albuquerque Junior R, Lourenco AP (2006). Ghrelin reverses molecular, structural and hemodynamic alterations of the right ventricle in pulmonary hypertension. Rev Port Cardiol.

[100] Xu X, Pang J, Yin H (2007). Hexarelin suppresses cardiac fibroblast proliferation and collagen synthesis in rat. Am J Physiol Heart Circ Physiol.

[101] Matsumura K, Tsuchihashi T, Fujii K, Abe I, Iida M (2002). Central ghrelin modulates sympathetic activity in conscious rabbits. Hypertension.

[102] Conraads VM, Hoymans VY, Vrints CJ (2008). Heart failure and cachexia: insights offered from molecular biology. Front Biosci.

[103] El Eter E, Al Tuwaijiri A, Hagar H, Arafa M (2007). *In vivo* and *in vitro* antioxidant activity of ghrelin: Attenuation of gastric ischemic injury in the rat. J Gastroenterol Hepatol.

[104] Iseri SO, Sener G, Saglam B (2008). Ghrelin alleviates biliary obstruction-induced chronic hepatic injury in rats. Regul Pept.

[105] Obay BD, Tasdemir E, Tumer C, Bilgin HM, Atmaca M (2008). Dose dependent effects of ghrelin on pentylenetetrazole-induced oxidative stress in a rat seizure model. Peptides.

[106] Zwirska-Korczala K, Adamczyk-Sowa M, Sowa P (2007). Role of leptin, ghrelin, angiotensin II and orexins in 3T3 L1 preadipocyte cells proliferation and oxidative metabolism. J Physiol Pharmacol.

[107] Ukkola O (2009). Ghrelin and metabolic disorders. Curr Protein Pept Sci.

[108] Vestergaard ET, Gormsen LC, Jessen N (2008). Ghrelin infusion in humans induces acute insulin resistance and lipolysis independent of growth hormone signaling. Diabetes.

